# Supplementary material for: Association between oral microbiome and seven types of cancers in East Asian population: a two-sample Mendelian randomization analysis
Source: Front Mol Biosci. 2023 Nov 21;10:1327893. doi: 10.3389/fmolb.2023.1327893 (PMC10702768; doi:10.3389/fmolb.2023.1327893)
Supplement: Supplementary file 1 [file DataSheet1.ZIP › Supplementary Materials/MR plots of saliva/saliva═╝/colorectal cancer/pheno.3599_to_colorectal cancer_forest.pdf]

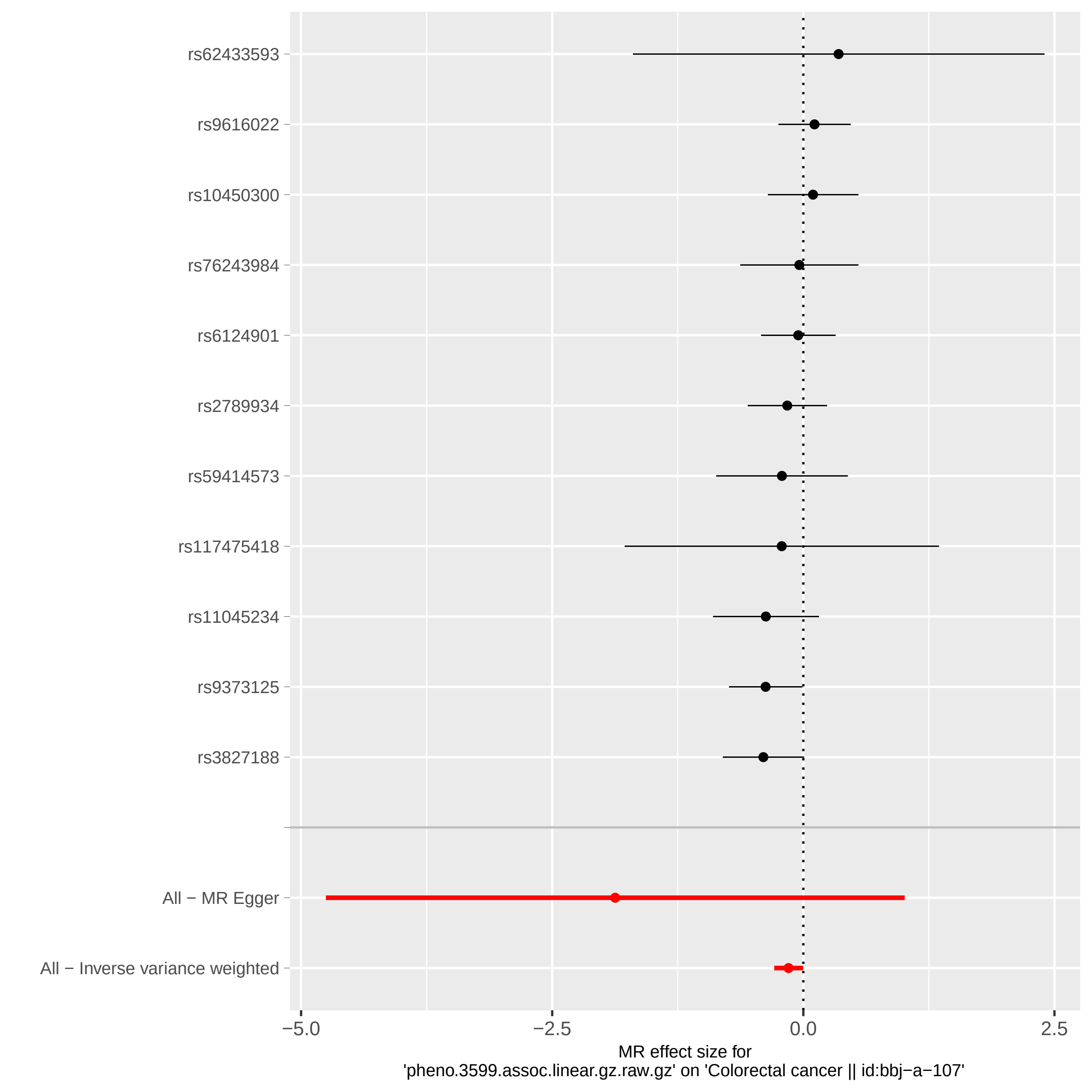
rs62433593
rs9616022
rs10450300
rs76243984
rs6124901
rs2789934
rs59414573
rs117475418
rs11045234
rs9373125
rs3827188
All − MR Egger
All − Inverse variance weighted
−5.0
−2.5
0.0
2.5
MR effect size for
'pheno.3599.assoc.linear.gz.raw.gz' on 'Colorectal cancer || id:bbj−a−107'
